# Simultaneous Second-Harmonic, Sum-Frequency Generation and Stimulated Raman Scattering in MgO:PPLN

**DOI:** 10.3390/ma11112266

**Published:** 2018-11-13

**Authors:** Dismas K. Choge, Huaixi Chen, Lei Guo, Guangwei Li, Wanguo Liang

**Affiliations:** 1 Fujian Institute of Research on the Structure of Matter, Chinese Academy of Sciences, Fuzhou 350002, China; cdismas2004@yahoo.com (D.K.C.); hxchen@fjirsm.ac.cn (H.C.); guolei@fjirsm.ac.cn (L.G.); gwli@fjirsm.ac.cn (G.L.); 2 University of Chinese Academy of Sciences, Beijing 100049, China

**Keywords:** lithium niobate, frequency conversion, phase-matching, visible light, Raman

## Abstract

In this study, simultaneous second-harmonic generation (SHG), sum frequency generation (SFG), and Raman conversion based on MgO-doped periodically poled lithium niobate (MgO:PPLN) for multi-wavelength generation is demonstrated. The approach used is based on a single MgO:PPLN crystal poled with a uniform period of 10.2 µm that phase matches SHG and SFG, simultaneously. Using a simplified double-pass geometry, up to 0.8 W of blue light at 487 nm is achieved by a frequency-doubling 974 nm laser diode pump, and 0.5 W of orange light at 598 nm is generated by frequency mixing 974 nm pump with C-band (1527–1565 nm) tunable laser source. At high pump powers of the 974 nm laser source, other unexpected peaks at 437, 536, 756, 815 and 1038 nm were observed, of which the 1038 nm line is due to Stimulated Raman Scattering within the MgO:PPLN crystal. The resulting multi-wavelength light source may find a wide range of applications in biomedicine and basic research.

## 1. Introduction

Visible light sources with diverse wavelengths have become indispensable for practical applications ranging from fundamental research to industry and entertainment. For instance, various fluorophores used in life science and biomedical research applications are known to excite at/or close to the 488 nm wavelength [1,2]. Likewise, orange light with wavelengths near 600 nm have found critical applications in basic science research and displays [3,4]. Such sources when based on MgO-doped periodically poled lithium niobate (MgO:PPLN) are very promising because of the combined advantages of high nonlinear coefficient and high damage threshold of this crystal [5]. Conventionally, frequency upconversion that comprise of second-harmonic generation (SHG) and sum frequency generation (SFG) are often used to generate hard-to-reach wavelengths in the visible spectral region enabled by means of quasi-phase-matching (QPM) technology. With the use of this technology, broadly tunable wavelength upconversion into the 770 nm wavelength region, based on chirped periodically poled lithium niobate (PPLN) structure, has been demonstrated [6]. In our previous work, we reported multi-wavelength upconversion into the visible range based on MgO:PPLN [7]. In addition, optical parametric oscillators (OPOs) employing resonant cavities are quite attractive for generating new wavelengths in-accessible through direct lasing in the visible range [8]. Such OPOs have found applications in high resolution Doppler-free spectroscopy, albeit, they are typically bulky [9]. An alternative double-pass approach has been shown to enhance the nonlinear conversion efficiency in periodically poled structures without stability problems associated with OPOs [10]. Moreover, higher pump powers in OPOs can lead to generation of new wavelengths attributed to Stimulated Raman scattering (SRS) in PPLN, and in MgO-doped periodically poled stoichiometric lithium tantalate crystals [11,12]. Optical nonlinear materials such as lithium niobate (LN) are known to possess increased concentration of Raman Scattering centers, and thus, they exhibit Raman conversion [13]. Despite polarized Raman Scattering spectra for LN having been examined previously [14,15,16], reports on Raman conversion in PPLN are currently limited. Additionally, SRS has been observed in a PPLN second-harmonic generator and in bulk KTiOPO_4_ (KTP) crystal employing a single-pass geometry [17,18]. However, there is no report on such phenomenon based on double-pass configuration in MgO:PPLN crystal.

In this work, we demonstrate simultaneous SHG and SFG processes in MgO:PPLN based on double-pass geometry. At high pump power, we observed stimulated Raman conversion of the pump (974 nm laser diode) in addition to other unexpected multiple wavelengths whose origin is admittedly unclear. Such a multi-wavelength light source has prospects for biomedical and basic research applications.

## 2. Experimental Configuration

**Figure 1:**
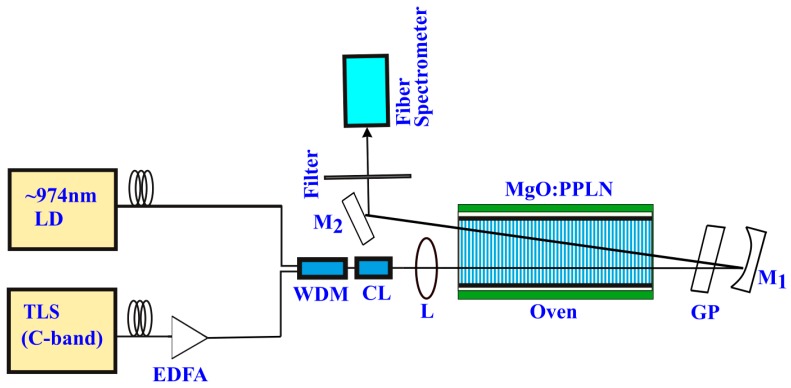
Schematic of the experimental setup for double-pass second-harmonic generation (SHG) and sum frequency generation (SFG) in a single crystal. TLS: tunable laser source; EDFA: erbium-doped fiber amplifier; LD: laser diode; WDM: wave division multiplexer; CL: collimator lens; L: lens; GP: glass plate; M_1_ and M_2_: mirrors.

The experimental setup for simultaneous SHG, SFG and SRS in MgO:PPLN involved a laser diode (LD) source emitting at 974 nm (*λ*_1_, 0.20 nm spectral width) serving as a pump (*P*_1_), and a C-band (1527–1565 nm) tunable laser source (TLS, *λ*_2_, <100 kHz linewidth) as a signal (*P*_2_) as shown in Figure 1. 

A detailed PPLN design is reported in our previous work [19]. Based on the design, a 50-mm-long MgO:PPLN crystal was fabricated using the standard electric poling technique [20], with a uniform grating period of 10.2 µm. A 1-mm-thick, z-cut, optical grade MgO:LN wafer was used. After poling, the crystal was cleaned and etched in a solution of hydrofluoric acid (HF), followed by end-face polishing and anti-reflective (AR, R < 0.2%) coating at 974 nm, 590–600 nm, and in 1525–1565 nm wavelengths. The signal light was amplified using EDFA, with a maximum output power of 2 W. The pump was combined with the amplified signal using a 50:50 wavelength division multiplexer (WDM) coupler. The collimated combined fundamental beam (using a C-lens CL) was focused into the 50 mm-long nonlinear MgO:PPLN crystal using a plano-convex lens L (f = 125 mm). The second-pass was realized using a highly reflective concave mirror (M_1_, R = 75 mm, coated for all the interacting wavelengths) with a slight incline, and consequently, the interaction period of the second-pass increased. The increased grating period causes a deviation from the optimum phase-matching resulting in a decrease in the conversion efficiency of the second-pass. An AR-coated glass plate (GP) placed between M_1_ and the crystal compensates for the phase shift resulting from dispersion in air [21]. This is accomplished by adjusting the GP, such that the net phase shift between the fundamental and the generated beams is a multiple of 2π. Another plano mirror M_2_ deflects the output beam for final evaluation. The average power incident on the MgO:PPLN crystal was measured to be *P*_1_ = 2 W and *P*_2_ = 0.8 W. The output power was measured with a power meter (Ophir-VEGA, Ophir-Spiricon, Inc., Jerusalem, Israel) and the spectra was detected with a fiber spectrometer (Resolution: 0.8 nm, BIM-6001, Brolight, Hangzhou, China). The wavelengths associated with SRS were eliminated using a set of two narrow-bandwidth filters centered at 600 and 490 nm for accurate SFG and SHG power measurements, respectively. The temperature of the MgO:PPLN crystal was regulated using a thermo-stabilized oven (model TCS-100, CTL photonics, Fuzhou, China) with 0.1 °C precision adjustable from 25 to 200 °C.

## 3. Results and Discussion

**Figure 2:**
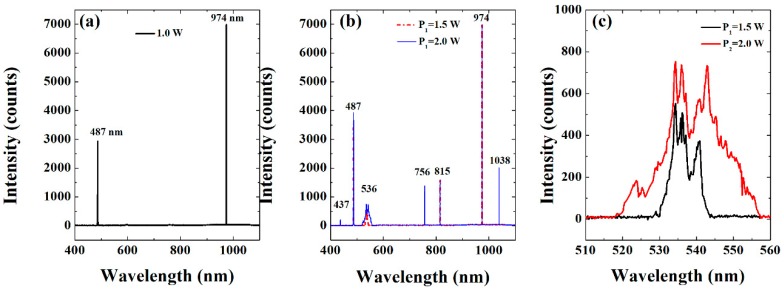
(**a**) Second-harmonic generation (SHG) spectrum of 974 nm source at 1 W power, (**b**) combined spectra of SHG, Raman conversion, and the unexpected lines at different pump powers, (**c**) Close-up view of the peak near 536 nm at different pump powers.

A series of spectra measured at different pump powers, is shown in Figure 2a–c. Figure 2a shows a pump and the corresponding second-harmonic spectra measured for 1 W of pump power. When the pump power increases, other unexpected peaks appear, one of which is due to Raman conversion in MgO:PPLN crystals as shown in Figure 2b. In particular, the peak at 1038 nm corresponds to a Stokes line of the 974 nm pump that can be assigned to transverse optical (TO_4_) phonon excitation mode with a Raman shift of 633 cm^−1^. This value of Raman shift is in close agreement with what was identified as the 8th Raman component in MgO:PPLN by Okishev and Zuegel [11], and to the TO_4_ mode reported in Reference [22]. The other observed peaks are at 437, 536, 756, and 815 nm. As for the 437 nm peak, we can presume its origin is a non-phase-matched sum frequency mixing of the 1038 nm and 756 nm wavelengths. However, it is not clear, at this point, whether the 536, 756, and 815 nm wavelengths are Raman or not. The frequency shifts for the 815 and 756 nm lines are 2003 and 2960 cm^−1^ relative to the pump wavelength, respectively, whereas the frequency shift between the 815 and 756 nm lines is 957 cm^−1^. None of these frequency shifts can be assigned to phonon modes reported for LN (see e.g., Reference [22]). This is also true for the 536 and 437 nm lines which give frequency shifts of 1877 and 2349 cm^−1^ relative to the 487 nm wavelength, respectively. Therefore, these phenomena require further investigation. Evidently, the number and intensities of the generated lines grow with pump power and at the same time, Figure 2c shows that the generated peak near 536 nm undergoes significant spectrum broadening with pump power, in agreement with previous observation in PPLN [23]. This broadening could come from self-phase-modulation (SPM) occurring as a result of refractive index nonlinearities induced by gain saturation in the fiber used to deliver the pump. The nonlinear refractive index coefficient $n_{2}$ of the fiber medium is intensity-dependent through the relation $n=n_{0}+n_{2}I$, where $n_{0}$ is the linear refractive index and I is the laser beam intensity [24]. Therefore, as the pump intensity changes, the refractive index of the fiber changes as well leading to intensity-dependent phase shift responsible for SPM. However, further investigation is still necessary to clarify the mechanism involved as no broadening was observed for other lines due to SPM.

**Figure 3:**
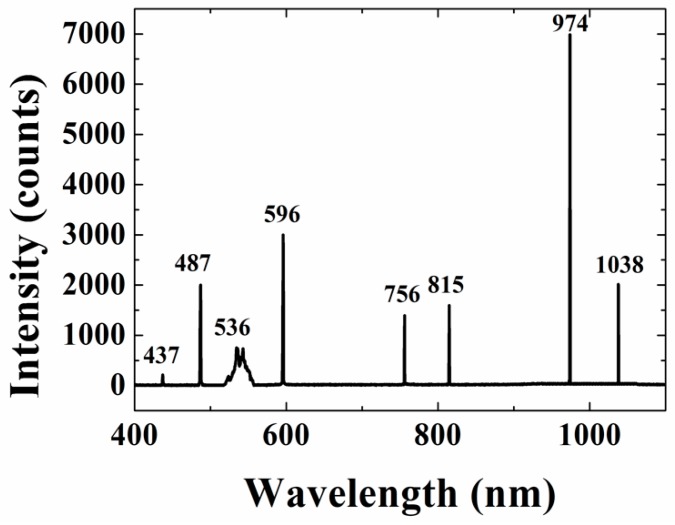
Spectrum of the combined second-harmonic generation (SHG), sum frequency generation (SFG), and Stimulated Raman Scattering (SRS) for full incident pump and signal powers at room temperature. Origin of the 536, 756, and 815 nm lines is unclear, but the 437 nm line could be a result of sum frequency mixing of the 1038 and 756 nm wavelengths.

In order to observe simultaneous SHG, SFG, and Raman operation, the TLS source was switched on. Figure 3 shows multiple peaks at 437, 487, 536, 596, 756, 815, and 1038 nm for the three simultaneous processes at room temperature while pumping with full input powers of the pump and signal. The decrease in the SHG intensity at 487 nm when the SFG process is started at 596 nm is mainly as a result of their competition since they both consume energy from the pump and the signal [25,26]. Again, it is unclear why there were no Raman lines observed near the 596 nm wavelength, but it is noteworthy that not all Raman components are generated at the operating wavelengths set by the grating period [27].

**Figure 4:**
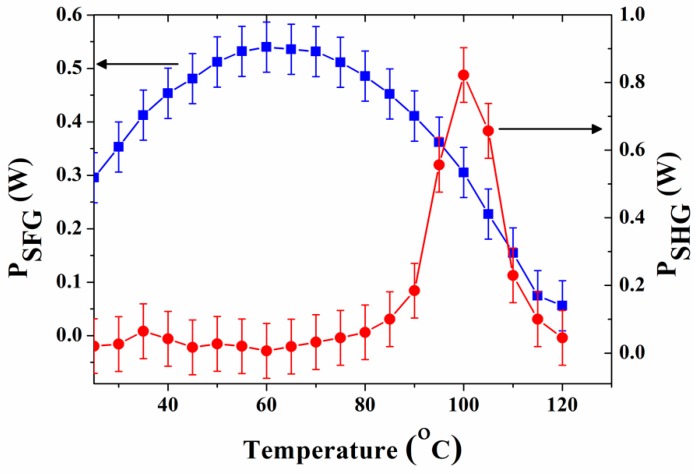
Temperature dependence of sum frequency generation (SFG) power *P_SFG_* (blue squares) and second-harmonic generation (SHG) power *P_SHG_* (red circles) at full incident pump and signal powers.

By tuning the MgO:PPLN crystal temperature from 25 to 120 °C, we noted that the Raman peak and the unexpected peaks become weak with an increase in the crystal temperature. This effect could be as a result of an increase in the SRS threshold with temperature rises. Figure 4 shows the observed variation of the generated SFG power (blue squares) and the generated SHG power (red circles) as a function of temperature.

**Figure 5:**
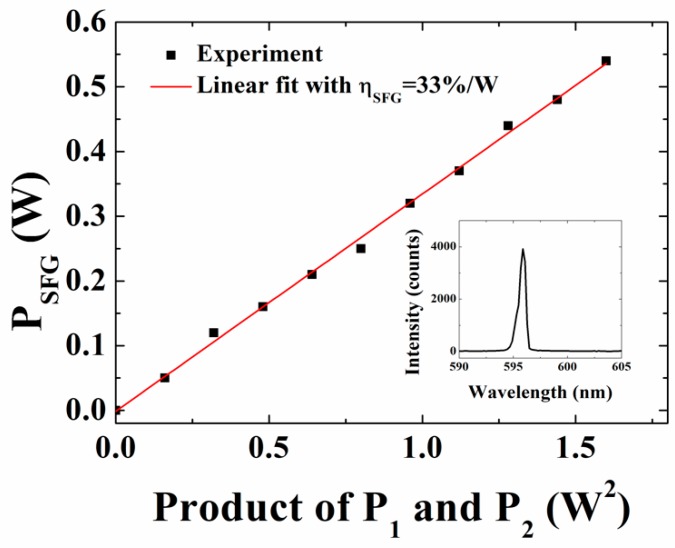
Sum frequency generation (SFG) power versus the product of pump (*P*_1_) and signal (*P*_2_) powers. The solid line is a linear fit to the SFG data using $P_{SFG}=\eta_{SFG}P_{1}P_{2}$. Inset: SFG spectrum at 598 nm measured at maximum incident powers of the pump and the signal.

At a crystal temperature of 60 °C, the SFG power reached its maximum, in agreement with the expected phase-matching temperature of the SFG process. We measured the generated SFG light at this temperature for various pump powers *P*_1_ up to a maximum value of 2 W by varying the operating current. Figure 5 shows the SFG power versus the product of pump (*P*_1_) and signal (*P*_2_) powers incident on the crystal for a fixed maximum (0.8 W) TLS power. A maximum SFG power was measured to be 0.5 W at 598 nm corresponding to SFG conversion efficiency of ~33%/W. The SFG peak position shifted apparently by ~2 nm (596–598 nm) since the phase-matching condition shifts to longer wavelengths with an increase in temperature. In contrast to the commonly used single-pass nonlinear conversion scheme, double-pass approach has been shown to enhance the conversion efficiency by an *N*^2^ factor for a fixed crystal length *L*, where *N* is the number of passes [10]. The measured conversion efficiency is, however, lower than the expected conversion efficiency for double-pass geometry of ~62%/W, estimated for full incident power (*P*_1_ = 2 W, *P*_2_ = 0.8 W) using the relation $\eta_{SFG}=P_{SFG}/P_{1}P_{2}$, where $P_{SFG}=32\pi^{2}d_{eff}^{2}P_{1}P_{2}L/n_{SFG}\varepsilon_{0}\lambda_{SFG}^{2}c(n_{1}\lambda_{2}+n_{2}\lambda_{1})$ [24]. Here, *c* is the speed of light in vacuum, $\varepsilon_{0}$ is the permittivity in vacuum, $d_{eff}=\left(2/\pi \right)d_{33}$, and $d_{33}$ for LN is ≈27 pm/V [28]. Furthermore, $n_{SFG}$(2.20) $n_{1}$(2.15), and $n_{2}$(2.13) are refractive indices at $\lambda_{SFG},\lambda_{1}$, and $\lambda_{2}$, respectively. The bracketed values were used in the calculation and can be obtained using the Sellmeier equation [29]. The observed low efficiency can partly be attributed to fabrication errors, non-uniform temperature distribution within the crystal, and poor focusing of the second-pass. Nevertheless, substantial improvement can be expected with optimized focusing and second-pass conditions. The inset in Figure 5 shows the corresponding SFG spectrum measured at 60 °C.

**Figure 6:**
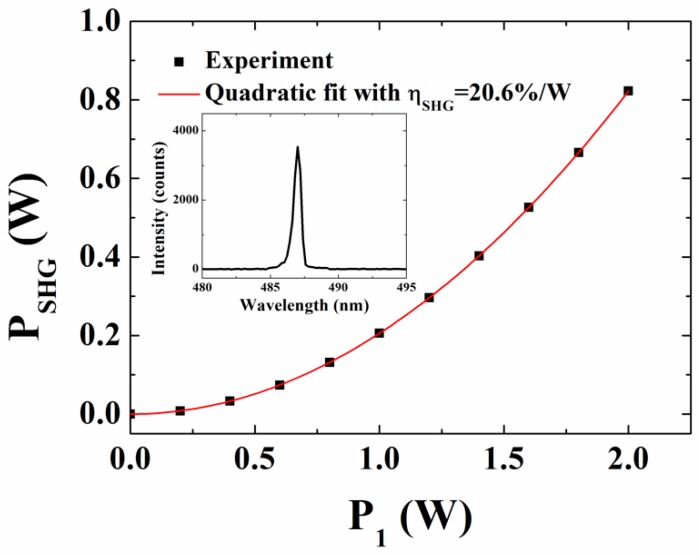
Second-harmonic generation (SHG) power versus the pump (*P*_1_) power. The solid line is a quadratic fit to the SHG data using $P_{SHG}=\eta_{SHG}P_{1}^{2}$. The inset is SHG spectrum at 487 nm measured at maximum pump power.

Further increase in temperature led to a maximum SHG power at 110 °C (red circles in Figure 4) which is the expected phase-matching temperature. Figure 6 shows the power of SHG light as a function of pump power. The SHG power varied to a maximum of 0.8 W at 487 nm measured at a temperature of 110 °C which corresponds to a conversion efficiency of ~20.6%/W for *P*_1_ = 2 W. The SHG conversion efficiency is defined as $\eta_{SHG}=P_{SHG}/P_{1}^{2}$ where $P_{SHG}=16\pi^{2}d_{eff}^{2}P_{1}^{2}Lh/n_{1}^{2}\varepsilon_{0}c\lambda_{1}^{3}$, and h is the Boyd and Kleinmann focusing parameter taking the value 0.8 for confocal focusing [30]. It is important to note that, QPM even-order processes such as the second-order SHG are possible since it is generally difficult to achieve a 50:50 duty ratio due to fabrication errors [31]. On the same note, the low SHG conversion efficiency is predominantly due to the second-order QPM SHG for the given domain period (10.2 µm). The conversion efficiencies could be further improved by using high fundamental power. The inset in Figure 6 shows the corresponding SHG spectrum measured at 110 °C.

## 4. Conclusions

In this study, simultaneous second-harmonic generation (SHG), sum frequency generation (SFG), and Stimulated Raman Scattering (SRS) in MgO-doped periodically poled lithium niobate with a single periodicity (ᴧ = 10.2 µm) is demonstrated. Using a double-pass configuration, up to 0.8 W of blue light at 487 nm is obtained by frequency-doubling a 974-nm laser diode pump, and 0.5 W of orange light at 598 nm is produced by frequency mixing a 974-nm pump with a C-band (1527–1565 nm) tunable laser source. We noted that increasing the pump power leads to unexpected peaks at 437, 536, 756, 815 and 1038 nm, one of which is believed to originate from SRS inside the MgO:PPLN crystal. Although the 1038 nm wavelength corresponds to a Stokes line of the 974 nm pump with a Raman shift of 633 cm^−1^, the origin of the lines at 536, 756, and 815 nm wavelengths remains to be determined, as they do not match the Raman shifts reported for lithium niobate, indicating that SRS is not responsible for their generation. Similar shifts for the 437-nm peak with respect to the 487-nm wavelength excludes its origin to SRS, but it could result from non-phase-matched sum frequency mixing of the 1038 and 756-nm wavelengths. Such a simultaneous, multi-wavelength light source may find promising biomedical and basic research applications.
